# P-40. Impact of case and control selection on influenza vaccine effectiveness (VE) among adults aged 40 years and older hospitalized with acute respiratory illness (ARI) during 2022-2023 using a test negative design (TND): secondary analysis of the North America multi-specimen study

**DOI:** 10.1093/ofid/ofae631.247

**Published:** 2025-01-29

**Authors:** Negar Aliabadi, Qing Liu, Julio A Ramirez, Allison McGeer, Ruth Carrico, Samira Mubareka, Sonal Uppal, Stephen Furmanek, Zoe Zhong, Thomas R Chandler, Caroline Kassee, Ashley M Wilde, Kevin Katz, Alan Junkins, Christie Vermeiren, Verna Welch, Malak Elsobky, Bradford D Gessner, Elizabeth Begier

**Affiliations:** Pfizer, New York, New York; Pfizer Inc., Collegeville, Pennsylvania; Norton Healthcare, Louisville, Kentucky; Mt. Sinai Hospital, Toronto, Ontario, Canada; Norton Healthcare, Louisville, Kentucky; Sunnybrook Health Sciences Centre, University of Toronto, Toronto, Ontario, Canada; Pfizer, New York, New York; Norton Healthcare, Louisville, Kentucky; Sinai Health System, University of Toronto, Toronto, Ontario, Canada; Norton Healthcare, Louisville, Kentucky; Sinai Health System, Toronto, Ontario, Canada; Norton Healthcare, Louisville, Kentucky; North York General Hospital, Toronto, Ontario, Canada; Norton Healthcare, Louisville, Kentucky; Shared Health Laboratories, Toronto, Ontario, Canada; Pfizer, New York, New York; Pfizer Canada, Toronto, Ontario, Canada; Pfizer Biopharma Group, Collegeville, Pennsylvania; Pfizer Vaccines, Dublin, Dublin, Ireland

## Abstract

**Background:**

Case and control definitions may impact VE estimates in observational studies. Using a TND design, we evaluated how influenza vaccine effectiveness against ARI hospitalizations changed when 1) influenza cases were detected through nasopharyngeal swabs (NPS) only versus multiple specimens and 2) controls included or excluded other vaccine preventable diseases (VPD).

**Figure:**
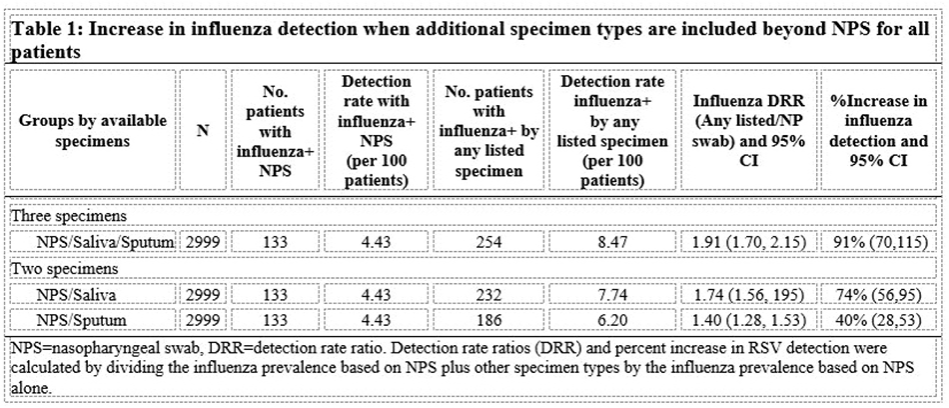

**Methods:**

Adults aged ≥40 years hospitalized for ARI had NPS and saliva or sputum tested using Luminex RSV/influenza multiplex PCR assay. Influenza was defined as either positive by NPS alone or positive by saliva, sputum, or NPS. Controls were defined as either all persons who were influenza negative or those negative for influenza and positive for respiratory syncytial virus (RSV). Exposure was self-reported seasonal influenza vaccination, defined as receipt of vaccine after 1 July 2022 and prior to 14 days of ARI illness onset. VE was calculated as 1 - ratio of odds of influenza vaccination among cases over controls.

**Figure:**
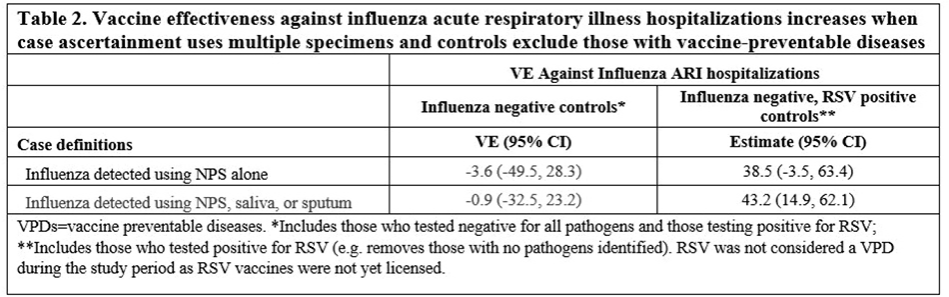

**Results:**

Among 2,999 patients with NPS and ≥1 other specimen, influenza detection increased by 40%, 74% and 91% when sputum, saliva, or both were added to NPS results, respectively (Table 1). Among 2,954 (98.5%) patients with influenza vaccine data, and when controls were defined as any influenza-negative ARI hospitalization, VEs were -3.6% (95%CI -49.5–28.3) and -0.9% (95% CI -32.5–23.2), using cases identified by NPS versus any specimen type, respectively (Table 2). When controls were persons who were RSV positive and influenza negative, VEs were 38.5% (95% CI -3.5–63.4) and 43.2% (95% CI 14.9–62.1) using cases identified by NPS versus any specimen type, respectively.

**Conclusion:**

Adding saliva and sputum nearly doubles influenza detection compared to NPS alone but does not substantially alter VE estimates. By contrast, VE was substantially increased when defining controls as those who were RSV+, yielding a result closer to the US Centers for Disease Control and Prevention values of 39-43% for the same season. The latter increase in VE may have occurred because RSV positivity reduces confounding caused by inclusion of other vaccine preventable infections (ie, SARS-CoV-2) in the control group due to correlation between vaccination against influenza and SARS-CoV-2.

**Disclosures:**

**Negar Aliabadi, MD, MS**, Pfizer Inc: employment|Pfizer Inc: Stocks/Bonds (Public Company) **Qing Liu, M.S.**, Pfizer Inc.: Stocks/Bonds (Public Company) **Julio A. Ramirez, MD, FACP**, Pfizer: Julio Ramirez is an employee of Norton Healthcare (Louisville, KY), which received fees from Pfizer in relation to this study. **Allison McGeer, MD**, AstraZeneca: Honoraria|GSK: Honoraria|Merck: Honoraria|Moderna: Honoraria|Novavax: Honoraria|Pfizer: Grant/Research Support|Pfizer: Honoraria|Roche: Honoraria|Seqirus: Grant/Research Support|Seqirus: Honoraria **Ruth Carrico, PhD, DNP, APRN**, Pfizer: Advisor/Consultant|Pfizer: Grant/Research Support|Pfizer: Honoraria|Sanofi: Advisor/Consultant|Seqirus: Advisor/Consultant **Samira Mubareka, MD**, Pfizer: Grant/Research Support **Verna Welch, PhD, MPH**, Pfizer Inc.: Stocks/Bonds (Public Company) **Malak Elsobky, MD**, Pfizer: Stocks/Bonds (Public Company) **Bradford D. Gessner, M.D., M.P.H.**, Pfizer: Employee|Pfizer: Stocks/Bonds (Public Company) **Elizabeth Begier, MD, M.P.H.**, Pfizer Vaccines: Employee|Pfizer Vaccines: Stocks/Bonds (Private Company)

